# Successful syllable detection in aphasia despite processing impairments as revealed by event-related potentials

**DOI:** 10.1186/1744-9081-3-6

**Published:** 2007-01-19

**Authors:** Frank Becker, Ivar Reinvang

**Affiliations:** 1 Sunnaas Rehabilitation Hospital, Faculty Division Ullevål University Hospital, University of Oslo, 0318 Oslo, Norway; 2 Dept. of Research, Sunnaas Rehabilitation Hospital, 1450 Nesoddtangen, Norway; 3 Dept. of Psychology, University of Oslo, 0317 Oslo, Norway

## Abstract

**Background:**

The role of impaired sound and speech sound processing for auditory language comprehension deficits in aphasia is unclear. No electrophysiological studies of attended speech sound processing in aphasia have been performed for stimuli that are discriminable even for patients with severe auditory comprehension deficits.

**Methods:**

Event-related brain potentials (ERPs) were used to study speech sound processing in a syllable detection task in aphasia. In an oddball paradigm, the participants had to detect the infrequent target syllable /ta:/ amongst the frequent standard syllable /ba:/. 10 subjects with moderate and 10 subjects with severe auditory comprehension impairment were compared to 11 healthy controls.

**Results:**

N1 amplitude was reduced indicating impaired primary stimulus analysis; N1 reduction was a predictor for auditory comprehension impairment. N2 attenuation suggests reduced attended stimulus classification and discrimination. However, all aphasic patients were able to discriminate the stimuli almost without errors, and processes related to the target identification (P3) were not significantly reduced. The aphasic subjects might have discriminated the stimuli by purely auditory differences, while the ERP results reveal a reduction of language-related processing which however did not prevent performing the task. Topographic differences between aphasic subgroups and controls indicate compensatory changes in activation.

**Conclusion:**

Stimulus processing in early time windows (N1, N2) is altered in aphasics with adverse consequences for auditory comprehension of complex language material, while allowing performance of simpler tasks (syllable detection). Compensational patterns of speech sound processing may be activated in syllable detection, but may not be functional in more complex tasks. The degree to which compensational processes can be activated probably varies depending on factors as lesion site, time after injury, and language task.

## Background

The analysis of speech sounds is a necessary step in the process of language comprehension. Since most aphasic patients have auditory comprehension deficits, the question whether and to what degree speech sound perception is impaired in aphasia has been much investigated [1-15]. Several studies have indeed shown that aphasic subjects perform significantly worse than healthy controls in e.g. tasks where they have to decide whether two consonants (or two syllables with different consonants) are the same or not [1,3,4,8,9].

However, most authors did not find correlations between these speech perception impairments and auditory comprehension abilities as measured by classical aphasia assessments [2,4,7,11]. Rather, several studies have revealed patients with severe auditory comprehension deficits but no or minor speech sound perception impairments, or patients with mild auditory comprehension deficits who performed poorly in speech sound discrimination and identification tasks [2-4,6,9,15]. Thus, a dissociation – at least partially – between speech perception and auditory comprehension has been found, which also has been quoted as evidence for a dual pathway framework of language comprehension [16]. However, a rather strong correlation between speech sound perception and auditory comprehension has also been reported [13].

Brain activity related to different stages of speech sound processing can be studied with event-related brain potentials. At about 100 ms after stimulus onset, a negativity can be recorded as the N1 wave which is generated in both temporal and frontal brain areas [17]. N1 reflects an intermediate stage in auditory analysis as well as sound detection and orienting functions [18]. Concerning the processing of speech sounds, N1 is suggested to reflect integrative processing of acoustic features of the incoming stream of speech, but not a neurological representation of phonemes [18-20].

Also the N2 waveform – recorded at about 150 to 300 ms after stimulus onset – is a summation of several components [21]. While early parts of the N2 (N2a or mismatch negativity, MMN) reflect automatic deviance detection, later stages of the N2 wave are regarded as correlates of attentional deviance detection (N2b) and of classification processing (N2c). Starting with N2b and in further stages, the processing of speech sounds seems to differ from that of non-speech sounds, while sound processing is common for speech and non-speech in earlier stages as reflected by N2a [22]. With regard to the time course of attentional discrimination of stimuli, it is suggested that the N2 component reflects processes of transient arousal triggered by unattended discrimination processes (reflected by N2a/MMN) which in turn trigger a target reaction [23]. Cognitive processes related to target detection and to the engagement of a target reaction are reflected by the P3 component which is mainly generated in parietal regions and in the case of auditory stimuli in superior temporal cortex [24-26].

Electrophysiological studies of sub-lexical speech sound processing in aphasia have mainly focused on unattended phonetic/phonologic processing often using the mismatch negativity component (MMN; for a short overview of these studies, see [27]). To our knowledge, no ERP-investigations of attended processing of sub-lexical speech stimuli have been performed in aphasia. While the number of studies using simple language stimuli in attended paradigms in order to investigate auditory processing is small, more studies with non-speech stimuli have been conducted, often using tones presented in oddball paradigms. There is good evidence for N1 amplitude reduction to an attended and frequent tone stimulus in aphasia [28-32]. Regarding topographic distribution of the N1 component, a right hemisphere maximum has been observed in an aphasic group while a control group showed an even hemispheric distribution [30]. Lesions located in either left or right superior temporal gyrus were found to be the cause for N1 amplitude reduction [33,34]. When using monaural presentation in left hemisphere injured patients, right-ear stimulation led to bilateral N1 reduction [35].

Regarding the response to the target stimulus, reduced P3 amplitudes have been reported, especially in patients with severe comprehension deficits [29,31,36]. The temporo-parietal junction has been shown to be crucial for normal P3 amplitudes to tone stimuli [37].

On the background of a still unclear relation between speech sound perception and auditory comprehension and sparse ERP-research on the attended processing of speech sounds in aphasia, we aimed in this study to further explore neurophysiological correlates of automatic and cognitive processes involved in speech sound processing in aphasic subjects. A major problem in interpreting ERP-results and behavioral findings is that when the study person fails to perform the task correctly, it is impossible to determine what underlying processes are active. Our strategy is therefore to study ERP in a relevant linguistic task which can be performed adequately by aphasic subjects, and to investigate the relevance of deviations in processing for the performance of a more complex task. Having investigated automatic discrimination of syllables in an earlier study [27], we used the same stimuli in this present study in an attended oddball design. A central research question was at which processing stages changes may be found in aphasia. Current research is focusing on changes of brain activation during recovery from brain injury, suggesting different activation patterns in patients with successful recovery compared to those with a less favorable outcome (e.g. [38]). Therefore, we grouped the participating patients with regard to aphasia severity. Furthermore, differences in topographic distribution of the components identified may give further information about functional or dysfunctional changes in brain activation, especially with regard to activation of ipsilesional and contralesional processes.

## Methods

### Subjects

A total of 20 aphasic subjects were consecutively recruited from patients admitted to our hospital for rehabilitation. 11 control subjects were recruited from hospital staff and non-brain damaged patients of the hospital. All participants with the exception of two severe and one moderate aphasic patient reported to be right-handed. Informed consent was obtained from all subjects. The study was approved by the regional research ethics committee of Eastern Norway.

All participants were examined with the auditory comprehension section of the Norwegian Basic Aphasia Assessment (NGA; [39]) and the Token test [40]. These tests measure comprehension in relation to both single words and short sentences, and with regard to both naturalistic objects, body parts, and geometric tokens. In addition, the patients were investigated with the complete NGA. Furthermore, all patients were assessed by a neuropsychologist as part of their routine rehabilitation program. Etiology and lesion location were retrieved from the patient's medical charts – the latter from descriptions of CT or MRI scans.

In order to investigate whether different electrophysiological patterns depend on the severity of the auditory comprehension deficit, the aphasic subjects were distributed into two groups: a group with aphasic subjects with mild or moderate auditory comprehension impairment (moderate aphasia group) and a group of subjects with severe or very severe auditory comprehension impairment (severe aphasia group). The parameter for dichotomization was a score of 16.5 in the shortened version of the Token test which corresponds to the border between moderate and severe aphasia as described by the authors [40].

Table 1 presents the patients with regard to sex, age, etiology, lesion site, aphasia type, language functions and neuropsychological impairments. The aphasic subjects represent a wide range of auditory comprehension impairment. Global and Wernicke's aphasia dominate the severe aphasia group, while anomia and Broca's aphasia were most common in the moderate aphasia group. In both groups, most patients have lesions in the frontal and/or temporal lobes; the most common cause for aphasia was brain infarction, but some more infrequent etiology was also present as for example traumatic brain injury or subarachnoid hemorrhage. Besides apraxia, neuropsychological impairments were mainly from the areas of attention, memory, executive and visual spatial functions.

**Table 1:** Demographical and clinical data of the aphasic subjects participating in this study.

**Severe aphasia group**									
**Sex**^a^	**Years of age**	**Type of aphasia**^b^	**Etiology**^c^	**Site of lesion**^d^	**Months post injury**	**Token test**	**NGA comp**^e^	**NGA total**^f^	**Areas of neuropsychological deficits**

F	53	GA	BI	FT	5,7	1	34	51	apraxia, visual spatial function
F	53	GA	BI	FTP	6,6	2	34	60	memory, visual spatial function
F	49	GA	SAH	FTP	2,5	4	13	35	apraxia, perseveration, problem solving, working memory
M	54	WA	BI	P	4,3	6	46	116	apraxia, executive function, abstract reasoning, visual attention, visual spatial function
M	59	GA	BI	FT	1,7	6.5	57	87	working memory, memory, problem solving
M	53	MTA	BI	P*	3,1	8	55	161	apraxia, memory, problem solving, visual scanning
F	48	GA	SAH, BI	F	4,7	10	54	163	attention, working memory, perseveration
M	45	GA	MS	n.d.	8,9	10.5	48	114	working memory, visual spatial function
M	67	WA	BI	FT*	3,6	11.5	28	107	executive function, problem solving, visual spatial function
F	55	WA	SAH	n.d.	97,7	11.5	51	n.d.	attention
									

**Moderate aphasia group**									

**Sex**^a^	**Years of age**	**Type of aphasia**^b^	**Etiology**^c^	**Site of lesion**^d^	**Months post injury**	**Token test**	**NGA comp**^e^	**NGA total**^f^	**Areas of neuropsychological deficits**

F	66	TSA	BI	FT	3,3	19	68	209	memory, visual spatial function
M	61	TSA	CH	FP	2,2	19.5	63	192	attention, executive function, visual discrimination
F	63	GA	BI	FT*	2,7	21	52	165	attention, executive function, memory, abstract reasoning
M	61	BA	BI	FTP	2,1	21.5	65	155	apraxia, attention, problem solving, visual spatial function
M	64	TSA	CH	PO	2,0	22.5	61	191	acalculia, apraxia, working memory, abstract reasoning, visual attention
M	41	BA	Tumor resection	F	0,8	23	66	185	working memory, visual spatial function
F	56	AA	TBI	FP	5,3	28	69	204	apraxia, memory
F	65	AA	Encephalitis	FT**	3,6	30.5	61	202	memory, visual attention
F	18	AA	TBI	F, T, P, O	3,5	30.5	69	209	attention, executive function, visual scanning and discrimination
M	36	AA	BI	P	20,6	32	70	n.d.	executive function

^a ^M = male; F = female

^b ^AA = anomic aphasia; BA = Broca's aphasia; GA = global aphasia; MTA = mixed transcortical aphasia; TSA = transcortical sensory aphasia; WA = Wernicke's aphasia

^c ^BI = brain infarction; CH = cerebral hemorrhage; MS = Multiple sclerosis; SAH = subarachnoid hemorrhage; TBI = traumatic brain injury

^d ^F = frontal; T = temporal; P = parietal; O = occipital

^e ^NGA comp = Norwegian Basic Aphasia Assessment, subsection auditory comprehension

^f ^NGA total = Norwegian Basic Aphasia Assessment, total score

* indicates patients with right hemisphere lesions.

** In addition, this patient had a small lesion in the right temporal lobe.

One-way analysis of variance (ANOVA) revealed significant differences (p < 0.001) between groups for all three clinical aphasia measures. Neither the severe nor the moderate aphasia group differed significantly from each other or the control group with regard to sex, age, years of education or time post injury (see table 2).

**Table 2:** Overview over the three investigated groups.

	**Severe (N = 10)**	**Moderate (N = 10)**	**Control (N = 11)**
**sex (female/male)**	5/5	5/5	6/5
**age (years)**	53.5 (45.1 – 66.9)	53.1 (18.0 – 66.0)	58.2 (33.0 – 74.1)
**education (years)**	12.4 (9 – 15)	14.2 (11 – 20)	13.8 (10 – 18)
NGA* aud. comprehension (0 – 71)	42 (13 – 57)	64 (52 – 70)	71 (71)
**NGA total (0 – 217)**	99 (35 – 163)	190 (155 – 209)	-
**Token test (0 – 36)**	7.1 (1 – 11.5)	24.8 (19 – 32)	33.6 (31 – 35)
**time post onset (months)**	4.5 (1.7 – 97.7)	3.0 (0.8 – 20.6)	-

No significant differences in sex distribution, age, years of education or time post onset were found. Mean values are reported, except for time post onset where median is used; minimum and maximum values in parentheses.

* = Norwegian Basic Aphasia Assessment

### Stimuli

The participants were presented with a syllable detection paradigm using the same natural speech sounds as in our earlier study of automatic syllable discrimination [27]: The frequent standard syllable /ba:/ (p = 0.85) and the infrequent target syllable /ta:/ (p = 0.15) were presented with a stimulus onset asynchrony of 1.5 s in a pseudo randomized order with the restriction that two targets could not follow each other (see additional file 1, a 30 s sample of the auditory stimuli). The syllables were digitally recorded from a female, middle-aged native speaker and cut and re-spliced at zero crossings of the steady-state vowel to obtain syllables of same length (/ba:/ = 245.9 ms; /ta:/ = 245.2 ms). The recordings of the syllables were low-pass filtered at 8 kHz. The syllables had rise/fall times of 20 ms. A total number of 205 syllables, amongst these 30 target syllables, were presented binaurally via headphones at approximately 80 dB SPL. The participants were seated comfortably in a rest chair or their wheel chair and were instructed to press a button with the index finger of their preferred hand as soon as possible when they heard the target syllable /ta:/. Since many of the subjects had severe comprehension deficits, the stimuli (up to 15 targets and 40 standards) were first presented without EEG-recording, and the subject's reaction was observed to assure that the participants had understood the task. Additionally, prior to the recordings for this present study all subjects had been presented for the same syllable stimuli in an unattended paradigm [27] in the same session.

### ERP-procedure

EEG was recorded continuously with a sample frequency of 500 Hz and an online band-pass filter with a range from 0.05 to 70 Hz at the following electrode sites: Fz, Cz, Pz, Fp1/2, F3/4, C3/4, P3/4, F7/8, T3/4, T5/6, O1/2, M1, and M2. A nose reference electrode was used. The continuous EEG-data were post-hoc analyzed using band-pass (1 – 15 Hz), zero-phase filtering and ocular artifact reduction using vertical oculograms [41]. Sweeps with amplitudes exceeding +/- 100 μV in any channel except of the vertical oculogram were excluded from the analysis.

The three left-handed participants had CT-verified right hemisphere lesions and left hemiparesis. For these participants, symmetrical and corresponding electrode labels were swapped between hemispheres. Thus, in this paper odd numbered electrode indices (F3, F7 ...) refer to the brain damaged hemisphere (normally the left) and even numbered electrode indices (F4, F8 ...) refer to the contralesional hemisphere. For the controls – all being right-handed – electrode labels of the left hemisphere are referred to as ipsilateral.

The standard syllable (/ba:/) waveforms were analyzed for the N1 component, the responses to the target syllable (/ta:/) for N1 and P3. For each group separately, mean peak latencies for the components were defined as the mean of the individual peak latencies located at maxima in the following time windows: N1 = 60 – 180 ms and P3 = 300 – 700 ms. Cz electrode was used to define the latencies for standard and target N1, while Pz was used for target P3. For each component respectively, time intervals were centered at the relevant group's mean peak latency to calculate mean amplitudes. These intervals had a duration of 30 ms for the N1 and 50 ms for the P3 component. Using the respective intervals which were derived by the above described procedure, mean amplitudes for the following electrode sites were calculated and further analyzed: Fz, Cz, Pz, F3/4, C3/4, P3/4, F7/8, T3/4, T5/6. A similar analysis was performed separately for the mastoid electrodes (M1/2); these results do not give additional information and are therefore not reported.

Furthermore, subtraction waveforms (target - standard) were analyzed to elucidate the process of discriminating targets from standards. Mean average amplitudes of successive time windows of 50 ms duration in the range from 75 ms to 475 ms were calculated and analyzed; this time span contains the N2 component.

### Statistical analysis

We analyzed the mean amplitudes using a two-way ANOVA model with the between subjects factor "group" (severe aphasia vs. moderate aphasia vs. control) and the within group factors anterior-posterior "line" (frontal vs. central vs. parietal) and "electrode" (5 levels; for example F7, F3, Fz, F4, and F8 for the frontal electrode line). Thus a significant interaction involving the "electrode" factor might indicate a hemisphere difference, but would have to be further analyzed focusing on the relevant electrode contrasts. Greenhouse-Geisser and Bonferroni corrections were applied when appropriate. Latencies were compared between groups using one-way ANOVAs.

Furthermore, Spearman's rank test was used to analyze ERP-amplitudes and latencies for correlations with time after brain-injury, reaction time (RT) and clinical aphasia assessment results (NGA auditory comprehension, NGA total, and Token test). Only aphasic subjects were included in these analyses, except for the RT-analysis where all participants were included. In order to reduce the risk of type I error – on the background of the large number of correlation analyses performed – the significance level for correlations was set to 0.01.

## Results

### Behavioral results

Almost all subjects detected all 30 targets; only three severe aphasic patients missed one target syllable each. Many participants had a few false alarms, but none more than four; no significant differences regarding false alarm rates were found. These results indicate that the task was a rather easy one.

The target response time was significantly prolonged in the patient groups (p < 0.05): While the mean reaction time was 383 ms in the control group, it was 465 ms in the moderate and 586 ms in the severe aphasic group.

### Standard syllable N1

**Figure 1:**
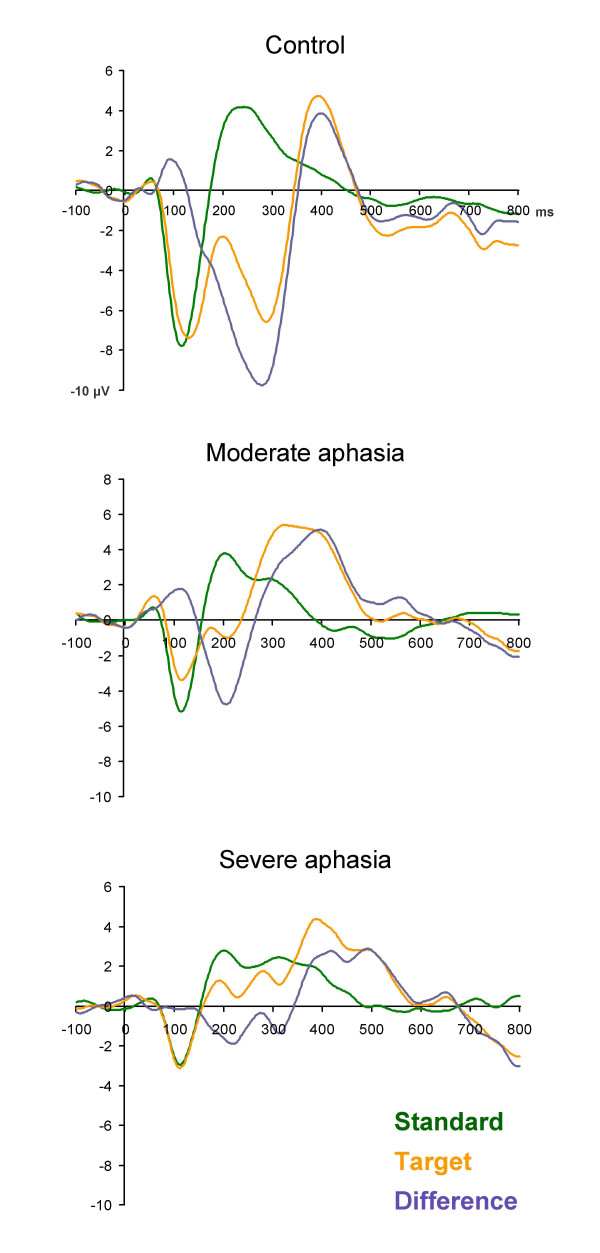
**Grand average waveforms**. Vertex grand average waveforms for the standard (green) and the target stimulus (orange) and the difference curve (blue grey) for the three groups respectively.

Grand average waveforms for the three groups respectively are presented in figure 1, mean amplitudes and standard deviations for selected electrodes in table 3.

**Table 3:** Mean amplitudes

**electrode**	**group**	**N1**	**N1 target**	**P3**	**N2 175–225 ms**	**N2 225–275 ms**	**N2 275–325 ms**
	severe	-0,16 (1,02)	-1,55 (1,82)	1,43 (4,39)	-1,41 (4,26)	-1,89 (4,32)	-1,98 (3,35)
**F3**	moderate	-2,44 (1,43)	-1,98 (2,16)	1,67 (2,58)	-0,46 (1,96)	0,35 (2,83)	1,61 (3,16)
	control	-3,18 (1,09)	-4,04 (1,35)	1,93 (2,99)	-3,21 (1,62)	-3,57 (3,07)	-2,34 (4,77)
	severe	-1,12 (1,26)	-1,35 (1,34)	3,11 (4,40)	-0,53 (1,94)	-0,31 (2,24)	-0,11 (3,63)
**Fz**	moderate	-3,22 (1,44)	-2,51 (2,23)	2,80 (3,31)	-0,82 (1,99)	-0,05 (3,09)	1,70 (3,11)
	control	-4,52 (1,39)	-4,92 (1,97)	2,60 (3,87)	-3,20 (2,14)	-4,84 (3,81)	-3,91 (5,13)
	severe	-0,76 (1,12)	-1,13 (0,97)	1,98 (3,48)	-1,41 (1,64)	-1,01 (3,07)	0,17 (3,87)
**F4**	moderate	-2,13 (1,72)	-2,14 (1,44)	2,36 (3,75)	-0,54 (1,45)	0,37 (2,59)	1,78 (3,55)
	control	-3,09 (0,65)	-3,54 (1,96)	2,14 (3,66)	-1,86 (2,90)	-3,26 (3,86)	-1,78 (5,46)
	severe	-1,13 (2,16)	-2,71 (3,43)	1,82 (3,45)	-2,61 (7,36)	-1,34 (5,78)	-1,68 (4,02)
**C3**	moderate	-3,76 (1,38)	-3,42 (3,16)	3,52 (3,48)	-3,82 (3,51)	-0,49 (4,46)	2,80 (3,31)
	control	-5,65 (2,02)	-7,05 (2,73)	2,12 (4,23)	-7,03 (3,46)	-6,87 (4,44)	-5,29 (5,96)
	severe	-2,54 (2,12)	-2,70 (2,49)	3,03 (4,10)	-1,60 (4,35)	-0,98 (4,25)	-0,94 (4,37)
**Cz**	moderate	-4,31 (1,30)	-2,96 (2,84)	3,94 (3,93)	-4,25 (4,64)	-1,33 (4,78)	2,51 (3,89)
	control	-7,01 (2,09)	-7,20 (3,11)	2,50 (4,20)	-5,56 (3,93)	-8,87 (6,86)	-8,28 (7,36)
	severe	-2,38 (1,96)	-3,17 (2,63)	2,89 (3,85)	-2,91 (2,83)	-2,24 (3,36)	-1,11 (4,57)
**C4**	moderate	-3,20 (1,74)	-3,39 (2,23)	3,63 (3,64)	-4,74 (3,62)	-2,47 (2,64)	1,40 (3,40)
	control	-5,45 (1,06)	-6,02 (2,45)	2,60 (3,94)	-5,60 (3,67)	-7,68 (5,08)	-5,93 (5,61)
	severe	-0,98 (2,20)	-2,20 (2,63)	2,06 (3,42)	-1,57 (2,79)	-0,85 (3,17)	-0,73 (3,76)
**P3**	moderate	-1,55 (1,10)	-1,50 (3,20)	3,47 (3,83)	-2,51 (3,26)	0,56 (3,55)	3,67 (4,51)
	control	-3,09 (1,25)	-4,02 (2,14)	3,63 (3,61)	-6,18 (3,69)	-7,25 (4,95)	-5,35 (6,38)
	severe	-1,79 (1,81)	-3,08 (2,61)	2,60 (4,08)	-1,45 (2,83)	-1,20 (2,88)	-0,71 (4,24)
**Pz**	moderate	-2,26 (1,18)	-2,14 (3,50)	4,58 (3,96)	-3,77 (4,10)	-0,76 (3,89)	2,90 (5,04)
	control	-4,37 (1,59)	-5,22 (2,48)	4,32 (4,25)	-6,08 (4,33)	-8,54 (6,43)	-6,89 (6,94)
	severe	-0,98 (1,59)	-2,61 (2,10)	2,83 (3,70)	-2,35 (2,42)	-2,33 (2,54)	-1,25 (4,50)
**P4**	moderate	-0,94 (1,00)	-1,49 (2,52)	3,38 (3,12)	-3,57 (3,59)	-1,04 (3,15)	1,65 (4,67)
	control	-2,92 (1,57)	-4,29 (2,51)	3,47 (3,81)	-6,14 (3,52)	-7,77 (4,98)	-4,92 (6,03)

Mean amplitudes and standard deviations (parentheses) for some selected electrodes for each group respectively. N1 elicited by standard and target syllable and P3 are shown; in addition those intervals from the subtraction wave where significant differences between groups were observed.

**Figure 2:**
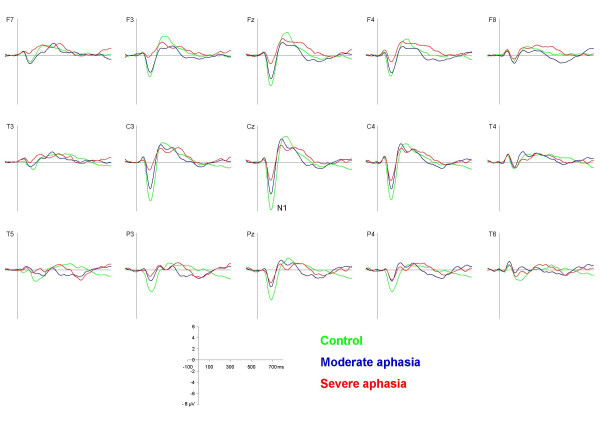
**Standard syllable waveforms**. Grand average waveforms elicited by the standard syllable /ba:/ for the control (green), the moderate (blue) and the severe aphasia group (red) respectively.

**Figure 3:**
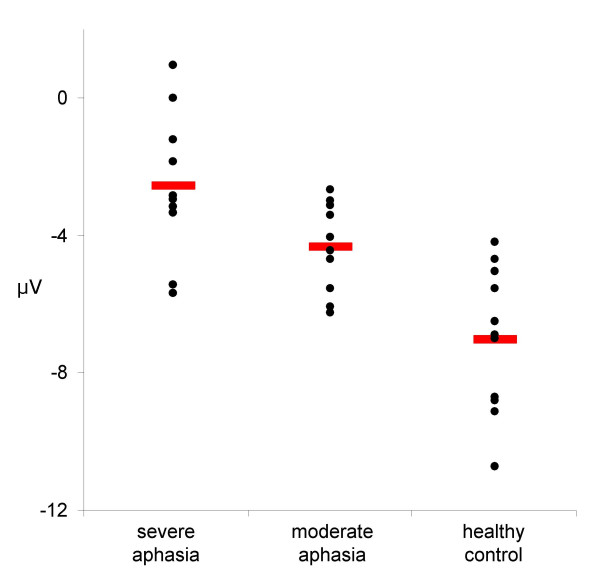
**Individual N1 mean amplitudes**. Scatter plot that shows the individual mean N1 amplitudes in μV (black dots) for each group separately, illustrating between-subject variation. Red bars indicate the respective group mean.

The N1 component was registered as a centrally peaking component with the following mean group latencies and amplitudes: control: 115 ms, -7.01 μV; moderate aphasia: 115 ms, -4.31 μV; severe aphasia: 110 ms, -2.54 μV (figure 2 and 3). The two-way ANOVA revealed a significant between groups effect (F [2.28] = 10.67, p < 0.001). Post-hoc analysis showed a significant difference between the control and the severe aphasia group (p < 0.001) and a marginally significant difference between the control and the moderate aphasia group (p = 0.053).

**Figure 4:**
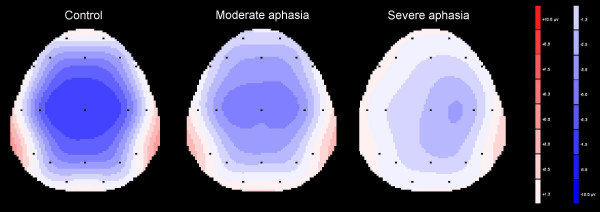
**Topographical distribution of the N1 component**. The control (left) and the moderate aphasia group (middle) show an even hemispherical distribution while the N1 of the severe aphasia group (right) is clearly lateralized to the contralesional hemisphere.

A significant line * group interaction was found (F [2, 28] = 3.15, p < 0.05). Analysis of each electrode line separately indicated that while the group differences were still present in all lines, the anterior-posterior N1 distribution varied. The mean N1-amplitudes in the controls were evenly balanced frontally and parietally, whereas the moderate aphasia group had larger amplitudes over frontal than parietal sites (F [1,9] = 6.40, p < 0.05) and the severe aphasia group showed a non-significant tendency for an inverse pattern. Furthermore, the two-way ANOVA revealed a highly significant electrode * group interaction: F [2, 28] = 8.20, p < 0.001, which reflected differences in hemisphere distribution of the N1 (figure 4). The controls had an even hemispheric N1 distribution (difference between corresponding electrodes < 0.2 μV) while the moderate aphasia group showed a minor relative lateralization (~0.4 μV) to the ipsilesional hemisphere and the severe aphasia group a distinct relative lateralization (~1.2 μV) to the contralesional hemisphere. This lateralization difference was most prominent in central areas. A tendency to a significant interaction line * electrode * group (F [2, 28]  = 2.00, p = 0.076) was observed for an analysis of the frontal and central line only. When using a hemisphere model with the electrodes F3/4 and C3/4, we found a significant hemisphere * group interaction (F [1,28]  = 3.38, p < 0.5).

### Target syllable N1

**Figure 5:**
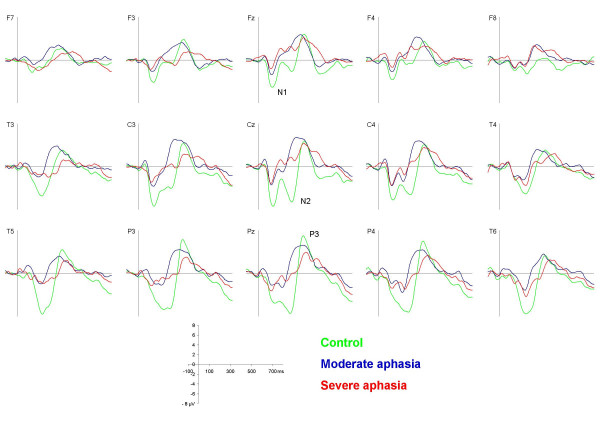
**Target syllable waveforms**. Grand average waveforms elicited by the target syllable /ta:/ for the control (green), the moderate (blue) and the severe aphasia group (red) respectively.

The N1 to the target syllable could be visually distinguished from the N2 component especially at frontal and central sites (figure 5). It peaked about 10 ms later than the N1 elicited by the standard syllable (127 ms, 124 ms, and 119 ms for the control, the moderate and the severe aphasia group respectively). The vertex amplitude of the target syllable N1 was comparable to that of the standard syllable N1 (see also table 3): -7.20 μV (controls), -2.96 μV (moderate aphasia), and -2.70 μV (severe aphasia).

Two-way ANOVA showed a significant between group effect (F [2, 28]  = 4.44, p < 0.05) which post-hoc analysis revealed to be significant for the control vs. moderate aphasia comparison (p < 0.05) and marginally significant for the control vs. severe aphasia comparison (p = 0.066). Further analysis of topographic anterior-posterior distributions showed the same tendencies as for standard-N1, but generally at a non-significant level. Visual inspection indicated a tendency towards the same hemisphere distribution differences as observed for the standard syllable elicited N1; a significant electrode * group interaction was found (F [2, 28] = 4.77, p < 0.001). The severe aphasia group showed larger amplitudes over the contralesional hemisphere especially at central and parietal sites.

### P3

The P3 component (figure 5, table 3) was observed in the controls as the typical large positivity with a parietal maximum peaking at 436 ms (4.32 μV). A somewhat earlier maximum was observed in the moderate aphasia group (peak: 419 ms, 4.58 μV). In the severe aphasia group, P3 was somewhat attenuated and peaked over the frontal midline (451 ms, 3.11 μV). However, no significant differences between groups in P3 mean amplitudes or latencies were found.

### Subtraction curve analysis

**Figure 6:**
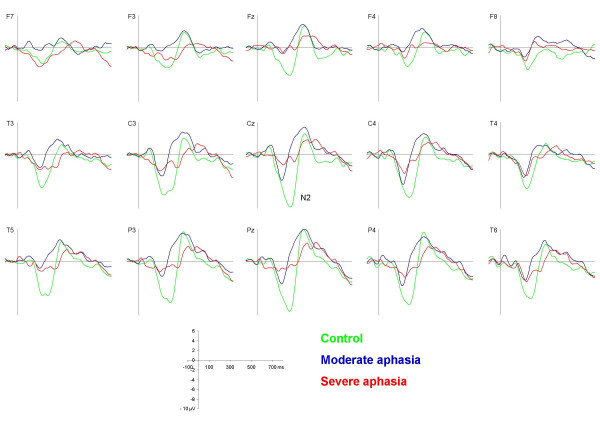
**Subtraction waveforms**. Grand average subtraction waveforms (target /ta:/ - standard /ba:/) for the control (green), the moderate (blue) and the severe aphasia group (red) respectively.

**Figure 7:**
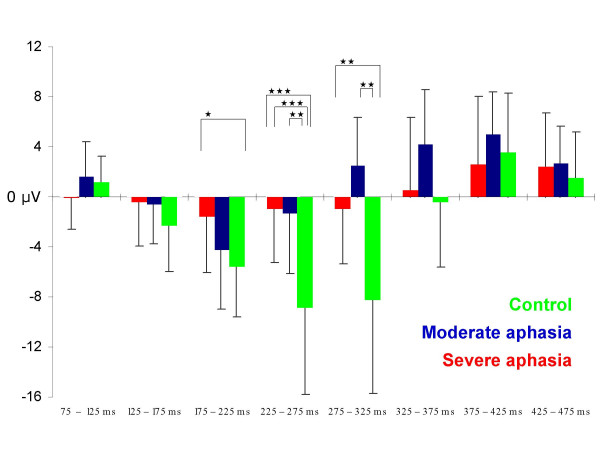
**Subtraction wave, mean amplitudes from time windows**. Mean subtraction amplitudes (target /ta:/ - standard /ba:/) at vertex illustrating the differences between the control (green), the moderate (blue) and the severe aphasia group (red) in time course and size of the N2 component. X-axis: 50 ms time-windows from 75 to 475 ms; y-axis: mean amplitudes in μV. Black bars indicate standard deviation (for graphical reasons only shown in one direction). Significant ANOVA between group effects are indicated: * p < 0.05, ** p < 0.01, *** p < 0.001.

The different time courses and distributions of the subtraction curves (target - standard waveform) for the three groups in successive 50 ms intervals in the time range 75 to 475 ms are illustrated in figures 6 and 7 (see also table 3). The negative processing difference of the control group started in the 125 – 175 ms window over left hemisphere temporo-parietal areas and developed into a large, central negativity that was registered over the whole scalp and lasting until about 325 ms. In the moderate aphasia group, the negative difference started in the same time window, but had a shorter duration and a more posterior and contralesionally centered maximum. The severe aphasia group showed low negative difference amplitudes at most electrode sites, but no clear lasting central negativity. Analysis of variance revealed significant differences between groups solely in the three time-windows between 175 and 325 ms which are described in the following.

*For the 175 – 225 ms interval*, the ANOVA showed a between groups effect (F [2, 28]  = 3.67, p < 0.05); post-hoc analysis resulted only in a tendency to significance for the control vs. severe aphasia group comparison (p = 0.062). A significant line * group interaction was found (F [2, 28] = 2.69, p < 0.05). Further analysis of the frontal line resulted in a tendency towards a between group effect (p = 0.089), while we observed a significant difference for the parietal electrodes (p < 0.05). The largest amplitudes at this stage were found parietally in the control group, but centrally in the aphasic groups. For the parietal line, we also found an electrode * group interaction (F [2, 28]  = 2.48, p < 0.05) with significantly different amplitudes between groups at P3, P4, and Pz electrode site. In this early segment of the processing difference, the control group's negativity was lateralized to the left hemisphere, whereas the moderate aphasia group showed higher amplitudes over the contralesional hemisphere at central and parietal sites.

The processing difference between target and standard stimulus in *the 225 – 275 ms time-window *increased – compared to the preceding interval – in the controls, but decreased in the aphasic groups. Analysis of variance showed a between groups effect (F [2, 28] = 10.80, p < 0.001) which was present between the controls and both the moderate (p < 0.01) and the severe aphasia group (p < 0.001). A significant line * group effect was found (F [2, 28]  = 4.42, p < 0.01). The processing difference of the control group was now centered between Cz and Pz electrode and centrally localized with regard to hemisphere distribution while it still showed larger amplitudes over the hemisphere contralesional to the brain damage in the moderate aphasia group.

*In the 275 – 325 ms interval*, the vertex mean amplitudes of the control and the severe aphasia group remained rather unchanged, while a positive amplitude indicated the start of a P3 effect in the moderate aphasia group. Also in this time-window the processing difference showed a between group effect (p < 0.01). Post-hoc analysis resulted in a significant difference between the control and the moderate aphasia group (p < 0.01). Line * group (F [2, 28]  = 3.37, p < 0.05) and electrode * group (F [2, 28]  = 3.44, p < 0.05) interactions were significant, and a significant line * electrode * group interaction was found (F [2, 28] = 2.24, p < 0.05), but the pattern of electrode differences did not indicate systematic hemispheric differences.

### Correlations ERP-parameters – clinical aphasia measures

Correlations between the results from the Norwegian Basic Aphasia Assessment (NGA), i.e. subsection auditory comprehension and the NGA total score, and ERP-components were found for mean amplitudes of the standard stimulus N1 in ipsilesional and midline fronto-central sites and for the mean amplitude of the 325 – 375 ms subtraction curve interval in left lateral parieto-temporal sites (table 4). Additionally we found tendencies for correlations (p < 0.05) at other fronto-central sites and also between the Token test and ipsilesional fronto-central electrodes.

**Table 4:** Overview of significant correlations

	**N1**		**Processing difference**		
	**standard**	**target**	**175 – 225**	**225 – 275**	**325 – 375**

**NGA auditory comprehension**	F3 (-0.66)				
	Fz (-0.59)				
	C3 (-0.63)				
**NGA total score**	F3 (-0.71)				T5 (0.63)
	Fz (-0.63)				
**Time post injury**	F3 (0.61)	M1 (0.69)	Cz (0.53)	Fz (0.47)	
	F7 (0.64)		C4 (0.52)	C4 (0.57)	

Overview of significant correlations between mean amplitudes (N1, processing difference) and auditory comprehension measures or time post injury. Spearman's correlation coefficients for the separate correlations are shown in parentheses. For all correlations: p < 0.01.

For the target stimulus N1, tendencies (p < 0.1) for correlations between the Token test and amplitudes at C3 and Cz electrode were observed, furthermore between M1 and the NGA total score.

### Correlations with reaction time

A positive correlation was found between P3 latency and reaction time (r_s _= 0.49, p < 0.01): the later the P3 component peaked, the longer was RT.

### Correlations ERP-parameters – time after brain injury

Moderate correlations between ERP-amplitudes and the time between brain injury and ERP-investigation were found for the N1 component elicited by the standard and the target syllable (table 4). Mean N1 amplitudes were smaller, the more time that had passed since brain injury.

## Discussion

In the present study, we investigated the ability of severe and moderate aphasic patients to detect rare target syllables amongst frequent standard syllables and studied the electrophysiological processes involved. The aphasic groups performed this rather easy task accurately, though more slowly than the controls. Despite the aphasics' successful task performance, we found several significant differences in their electrophysiological processing indicators. No alterations in ERP latencies were observed, but changes in ERP amplitudes for components found in the time range from about 100 and up to about 300 milliseconds after stimulus onset indicate differences during on-line stimulus processing or immediately following. These changes were primary stimulus processing reduction in the form of attenuated N1 amplitude for both standard and target stimuli at a latency of about 110 to 120 milliseconds, and a discrimination deficit between targets and standards in the time interval between 175 to 325 ms post stimulus onset. In this time range a clear N2 peak could be identified in the controls, whereas the aphasics showed a less distinct negative processing difference. P3 latency or amplitude did not differentiate between the groups, but was associated with reaction time. N1 amplitude reduction at ipsilesional fronto-central sites correlated with severity of auditory comprehension impairment. In addition, N1 amplitude at fronto-central electrode sites was smaller with increasing time after injury.

Topographic analysis indicated that moderate and severe aphasics showed different patterns of brain activation in order to solve the discrimination problem. Salient differences were that the severe aphasics showed a lateralization of activity focus to the contralesional hemisphere in an early processing window (N1), while showing no evidence of discriminatory activation in later time windows. The moderate aphasics on the other hand showed a more symmetrical activation in the corresponding early time window with evidence of discriminatory activity in later time windows. The implications will be discussed further below.

The observed attenuation of the N1 component in the aphasic groups is consistent with earlier findings for tone [29-34,36] and word stimuli [28,42]. A statistical correlation between N1 amplitude and measures for the severity of auditory comprehension measurement in aphasia has not been reported earlier, but in two studies that also dichotomized the aphasic patient groups in relation to auditory comprehension function, a larger N1 reduction in the severe aphasia groups has been reported [36,43].

N1 reduction and its correlations with auditory comprehension impairment can be interpreted as impaired sound detection and orienting functions and deficient integration of the acoustic properties of speech sounds [18]. Reduced N1 amplitude was found for both the standard and the target syllable which argues for a disturbance of primary stimulus processing independent of the role of the stimulus in the task. This is supported by the fact that the discrimination analysis (subtraction wave) did not reveal differences between groups in the N1 time window, but starting after 175 ms.

The deviant electrophysiological patterns in the aphasic groups between 175 and 325 ms argue for disturbances in the processes of attentional detection of the infrequent syllable /ta:/ and of its classification as the target stimulus. These differences were found in temporal stages of the N2 waveform which have been identified as being different between speech sound and purely acoustic processes [22].

However, the P3 component was not significantly altered in the aphasic groups indicating no severe impairments of target detection and processes of engaging the target reaction; this of course corresponds to the fact that the aphasic patients were able to detect the target syllables behaviorally. The lack of a significant P3 reduction – which contrasts some results of earlier P3 studies of aphasic patients – might be due to the large difference between the stimuli and to the relatively low difficulty of the task. In earlier studies, the stimuli were rich tones differing in only one parameter: frequency [29,31,36,37] or duration [37]. Actually, the reported P3 attenuation in the aphasic groups in most of these studies [29,31,36] was not caused by a general processing defect in aphasia, but rather – as the authors noted – by the fact that several subjects were unable to perform the task; in this present study, even the very severe aphasic subjects were able to accomplish the task almost without errors.

The close relation between the P3 component and the target response was illustrated by a significant, though weak correlation between P3 latency and reaction time. Although reaction time was significantly prolonged in the aphasic groups, we did not observe P3 latency differences between groups. This might be explained by disturbances in "post-P3" executive motor functions in the aphasic subjects, many of whom had sensory-motor deficits involving the preferred hand.

How can it be explained that the aphasic subjects were able to perform the current task successfully at the same time as the electrophysiological parameters are significantly attenuated and even correlate with auditory comprehension measures? A possible suggestion is that stimulus discrimination in at least some aphasic subjects was based not on linguistic analysis, but only or mainly on purely acoustic features. This strategy is adequate in a task with a very limited set of stimuli and no demands on semantic interpretation, but is not functional in a naturalistic comprehension task. Earlier studies have indeed shown that the ability to discriminate phonemes is a necessary, but not sufficient condition for the correct identification of these phonemes, and report several aphasic subjects that could discriminate, but not identify speech sounds [5,15]. We would argue that the severe aphasia group, which showed the largest N1 amplitude reduction, has to rely primarily on acoustic analysis. Linguistic processing – which accounts for parts of the N1 and a more substantial part of the N2 waveform – might thus be reduced in these subjects even if these linguistic analyses were not necessary to perform the task correctly. In this perspective, one could furthermore argue that speech sound discrimination based on purely acoustic features requires more resources and is more exhausting than "normal" speech sound discrimination; this could be suggested as one reason why aphasic subjects often report that listening to language is fatiguing (cf. [44]).

There are some other possible reasons for the observed amplitude reductions. First, compensational pathways might exist in aphasic brains which are not revealed by ERPs, at least not as recorded in the present study. These might be processes asynchronous in relation to the stimuli. Alternatively, the N1 and N2 components in healthy subjects might (partially) be generated by unnecessary, redundant activity that can be reduced in brain injured individuals without having impact on brain functions. Also, one could question the usually proposed sequential nature of the processing steps reflected by the N1, N2, and P3 components: Rather, different processes might exist in parallel. In injured brains, due to a conflict of resources, early processing steps might then be reduced because task-relevant processes are ongoing and prioritized.

However, an important objection to these interpretations of the present results is that the observed electrophysiological changes might not be due to impaired language functions, but rather solely to deficits in purely acoustic processing. On the other hand, one could argue that the amplitude attenuations might be only unspecific effects of brain lesion and lesion size which are not related to aphasia in particular. These problems can be addressed in a study using both a speech sound paradigm and a paradigm with purely acoustic stimuli, and furthermore by comparing aphasic patients with brain injured individuals without aphasia. We are pursuing this approach in an ongoing study.

Some interesting changes in the hemispherical distribution of brain activity were observed: As N1 maximum was located with an even hemispheric distribution in the controls, the aphasic groups showed two contrasting patterns of N1 hemisphere distribution at fronto-central sites: in the moderately impaired aphasic subjects, N1 was evenly distributed or even slightly lateralized to the ipsilesional side while it had more relative weight over the non-brain damaged hemisphere in patients with severely impaired auditory comprehension. Similar to the results regarding the severe group, relatively enlarged N1 amplitudes at contralesional fronto-central sites have been reported [30,42]. In a study using monaural stimulation, a similar pattern was found only for right-ear, but not for left-ear stimulation [35].

These findings might be explained by the effect of two different, but interacting mechanisms: First, a general N1 reduction takes place which is directly caused by the brain damage and which is larger in those patients with larger brain lesions and more severe impairments, i.e. the severe aphasia group. This attenuation is probably largest over brain damaged areas. Second, different compensational mechanisms in response to the brain damage might exist: Severely impaired patients activate the contralesional hemisphere relatively more than the ipsilesional hemisphere, while patients with lesser impairment show higher activation of the brain damaged than of the contralesional hemisphere. Thiel et al [38,45,46] have reported similar lateralization differences between patients with moderate aphasia and those with more impaired language function and claim a hierarchy of language recovery where the compensational activation of perilesional areas leads to rather good results, while the contralesional hemisphere can be activated as part of a less efficient compensational mechanism. Our results regarding the N1 component support this hypothesis, and we note that the majority of significant correlations between auditory comprehension score and single electrode N1 amplitudes are with ipsilesional fronto-central electrodes.

The ability to make use of compensational strategies in speech sound processing probably differs between aphasic subjects due to factors as premorbid brain organization and lesion site and size, but also depending on features of the speech sounds that are processed. This variation might be a reason for the complex relation between impaired speech sound perception and auditory comprehension in aphasia.

The clinical use of event-related brain potentials in order to explore and possibly monitor auditory comprehension in aphasia is under discussion [47-50]. The present study supports the usefulness of event-related potentials in the investigation of processes underlying auditory comprehension deficits in aphasia. As this study indicates, ERPs provide information about central auditory processing deficits even in tasks which are successfully accomplished by the aphasic subjects. Our results regarding the N1 and N2 waveforms – particularly the significant correlations of N1 amplitudes with clinical language comprehension assessment results – suggest that these waveforms deserve further attention in the exploration of auditory comprehension impairment in aphasia.

## Conclusion

This study investigated attended speech sound processing in aphasia recording event-related potentials during a syllable detection task. The aphasic subjects were able to perform the task almost without errors, and processes related to the target identification (P3) were not significantly attenuated. However, electrophysiological components reflecting primary stimulus analysis (N1) and attended stimulus classification and discrimination (N2) indicated reduced processing, which constitutes a crucial weakness in more complex and naturalistic comprehension tasks. The aphasic subjects might have discriminated the stimuli by increased reliance on acoustic differences, and topographic differences between aphasic subgroups and controls indicate compensatory changes in activation. The degree to which compensational patterns of speech sound processing can be activated probably varies depending on lesion site, time after injury, and language task.

## Competing interests

The author(s) declare that they have no competing interests.

## Authors' contributions

The study was designed and planned by FB and IR. FB carried out the data acquisition. Statistical analysis was performed by FB under the supervision of IR. The manuscript was drafted by FB. Both authors read and approved the final manuscript.

## Supplementary Material

Additional file 1 **Speech sound stimuli**. A 30 s sample of the task paradigm. Click here for file
